# Diphenyl diselenide derivatives inhibit microbial biofilm formation involved in wound infection

**DOI:** 10.1186/s12866-016-0837-x

**Published:** 2016-09-21

**Authors:** Luca Sancineto, Miranda Piccioni, Stefania De Marco, Rita Pagiotti, Vanessa Nascimento, Antonio Luiz Braga, Claudio Santi, Donatella Pietrella

**Affiliations:** 1 Department of Pharmaceutical Sciences, University of Perugia, Via del Giochetto, 06122 Perugia, Italy; 2 Laboratorio de Sıntese de Substancias de Selenio Bioativas, Centro de Ciencias Fısicas e Matematicas, Departamento de Quımica, Universidade Federal de Santa Catarina, 88040-900 Florianopolis, SC Brazil

## Abstract

**Background:**

Organoselenium compounds have antimicrobial activity against some bacteria and fungi; furthermore, the antioxidant activity of diselenides has been demonstrated. The aim of the present work was to examine the in vitro minimal inhibitory concentration of a panel of differently substituted diselenides and their effectiveness in inhibiting biofilm formation and dispersing preformed microbial biofilm of *Staphylococcus epidermidis, Staphylococcus aureus, Streptococcus pyogenes* and *Pseudomonas aeruginosa* and the yeast *Candida albicans,* all involved in wound infections. Moreover, the cytotoxicity of the compounds was determined in human dermal fibroblast and keratinocytes. In closing, we tested their direct antioxidant activity.

**Results:**

Diselenides showed different antimicrobial activity, depending on the microorganism. All diselenides demonstrated a good antibiofilm activity against *S. aureus* and *S. epidermidis*, the compounds camphor diselenide, bis[ethyl-N-(2’-selenobenzoyl) glycinate] and bis[2’-seleno-N-(1-methyl-2-phenylethyl) benzamide] were active against *S. pyogenes* and *C. albicans* biofilm while only diselenides 2,2’-diselenidyldibenzoic acid and bis[ethyl-N-(2’-selenobenzoyl) glycinate] were effective against *P. aeruginosa*. Moreover, the compounds bis[ethyl-N-(2’-selenobenzoyl) glycinate] and bis[2’-seleno-N-(1-methyl-2-phenylethyl) benzamide] showed an antioxidant activity at concentrations lower than the 50 % of cytotoxic concentration.

**Conclusions:**

Because microbial biofilms are implicated in chronic infection of wounds and treatment failure, the combination of antimicrobial activity and potential radical scavenging effects may contribute to the improvement of wound healing. Therefore, this study suggests that bis[ethylN-(2’-selenobenzoyl) glycinate] and bis[2’-seleno-N-(1-methyl-2-phenylethyl) benzamide] are promising compounds to be used in preventing and treating microbial wound infections.

## Background

Common etiologic agents of wound infection are *Staphylococcus* and *Streptococcus* species, *P. aeruginosa* and *Enterococcus* species [1]. Both acute and chronic wounds are sensitive to bacterial infection. Obesity is increasing worldwide; it is often associated with diabetes and complications such as chronic venous leg ulcers and diabetic foot ulcers. The prevalence of diabetes mellitus is estimated to be more than 371 million people worldwide and the number of diabetic patients is increasing everywhere [2]. Moreover, pressure ulcers, localized injuries of the skin usually in proximity of bony prominences, are a serious problem for all bed-bound and chair-bound patients [3]. Wound infections may also occur in burn victims [4], patients with traumatic wounds [5], and patients with surgical wounds [6]. The wound environment facilitates the development of microbial communities often associated in biofilms. Biofilms are microbial sessile communities in which microorganisms live attached either to abiotic or biotic substratum or to each other, in a matrix composed of proteins, lipids and polysaccharides, where they are more resistant to antimicrobial drugs and immune system responses with respect to the planktonic form [7]. Biofilms are found on the surface of the skin and a considerable amount of evidence suggests their involvement in the delay of wound healing and in the chronic inflammation process [8]. The prevention of biofilm formation is the goal of wound treatment because the standard protocols based on topical and systemic administration are often unable to remove biofilms. In fact, a regular debridement of chronic wounds is the main tool for maintaining a healthy wound bed [9].

Selenium (Se), in the form of selenoproteins or small organoselenium derivatives, is involved in numerous redox equilibrium and redox processes in living systems [10–12] and it is known to catalyze the formation of superoxide radicals which are able to inhibit the attachment of microorganisms to the solid surface [13]. Many studies have demonstrated the antimicrobial activity of different organoselenium compounds [14]. Diselenides have been studied for their antimicrobial activity; diphenyl diselenide and 2,2’-dithienyl diselenide have shown antibacterial and antifungal acivities [15, 16], moreover, selenocyanate and diselenides have been considered as a new class of antileishmanial compounds [17]. Finally, alkyl and aryl diselenides have shown microbial and antiviral activities [18].

In this study, we analyzed the ability of a panel of differently substituted diselenides whose characteristics influence the formation and dispersal of microbial biofilm of *S. epidermidis, S. aureus, S. pyogenes, P. aeruginosa* and the yeast *C. albicans* in different ways. These microorganisms are all involved in wound infections and frequently display drug-resistance, becoming a serious obstacle in acute and chronic wound treatment. Moreover, we tested the cytotoxicity of diselenides in human fibroblasts and keratinocytes and their radical scavenging activity.

## Methods

### Diselenides

All the diselenides used in the study are summarized in Table 1. Diphenyl diselenides (9) is commercially available by Sigma Aldrich; all the other compounds were synthesized with procedures reported in literature starting from antranilic acid (10, 10_d_, 10_e_) [19], the nicotinic acid (10_c_) [20] and camphor (11) [21]. All compounds were dissolved in methanol and stock solutions, at a concentration of 10 g/L; they were stored in the dark at −20 °C.

**Table 1:** Diselenides used in the study

Compounds	Formula	Molecular weight	Abbreviation used in the text
*Diphenyl diselenide (PhSe)*_*2*_		312	9
*2,2’-diselenidyldibenzoic acid DSBA*		402	10
*Camphor diselenide*		332	11
*2,2’-diselenidyldinicotinicic acid*		404	10_c_
*Bis[ethyl N-(2’-selenobenzoyl)glycinate]*		570	10_d_
*Bis[2’-seleno-N-(1-methyl-2-phenylethyl)benzamide]*		634	10_e_

### Microbial strains and growth conditions

The microbial strains used in this study were the four Gram-positive bacteria *Staphylococcus aureus* (ATCC 29213), *Staphylococcus epidermidis* (ATCC 35984), *Streptococcus pyogenes* (ATCC20565), *Streptococcus pneumoniae* (ATCC 20566), the Gram-negative *Pseudomonas aeruginosa* (ATCC 15692) and the yeast *Candida albicans* (SC5314). The bacterial cultures were maintained in tryptic soy agar (TSA). The day before the test, one colony was inoculated in tryptic soy broth (TSB) and incubated for 24 h at 37 °C. *Candida* cells from stock cultures in Sabouraud agar with 50 μg/ml chloramphenicol were grown in Sabouraud broth at 37 °C for 24 h. Microbial cells were harvested by centrifugation, washed, counted by spectrophotometric analysis and resuspended to the desired concentration in the appropriate culture medium.

### Determination of minimum inhibitory concentration (MIC)

MICs against microbial strains were determined by broth microdilution using two-fold serial dilutions in Muller Hinton Broth for bacteria and RPMI 1640/MOPS for *C. albicans* as described by the Clinical and Laboratory Standards Institute (CLSI) method. The test was carried out in 96-well U-bottom microdilution plates. Microbial inocula were prepared by subculturing bacteria into Muller Hinton Broth (MHB) and *Candida* cells in Sabouraud Broth at 37 °C for 18 h and then diluted to approximately 10^5^–10^6^ CFU/ml in MHB or RPMI/MOPS. One hundred μl of test compounds were diluted 1:2 in appropriate medium and placed in a 96-well tissue culture plate. The initial concentrations of the compounds used was 250 mg/L. One hundred μl aliquots of test microorganisms were added to each well. Microplates were then incubated at 37 °C for 24 h. Each experiment was repeated at least three times. As positive growth control, wells inoculated with microorganisms in the absence of the tested compound were carried out. MIC value was defined as the lowest concentration of compound that inhibits microbial growth. The positive control for Gram-positive and Gram-negative bacteria was gentamicin, and fluconazole for *C. albicans*.

### Growth curve inhibition

The antimicrobial activity of promising compounds against Gram-positive bacteria was investigated on the basis of MIC values (2xMIC, 1xMIC, 0.2xMIC). Tests were carried out in a 96 well culture plate. Two hundreds μl of microbial suspensions in MHB (10^5^ cells/ml) were incubated at 37 °C in a microplate reader (Infinite 200 pro, TECAN). From time 0, the absorbance (600 nm) of the culture was evaluated every 30 min for a total of 18 h. Results are presented as the mean of absorbance. Each analysis was performed in triplicate.

### Effect of diselenides on biofilm formation

The in vitro static biofilm assay was performed using a 96-well microtiter plate, as previously described, with some modification [22]. Bacteria were grown in TSB overnight. To cultivate biofilms, the overnight cultures of tested microorganisms were diluted 1:100 in fifteen ml of growth medium (TSB supplemented with 2 % sucrose) in the presence or absence of the different diselenides tested at the concentrations indicated. The positive control for Gram-positive and Gram-negative bacteria was gentamicin and fluconazole for *C. albicans*. Cultures were incubated at 37 °C for 24 h in static conditions. After incubation, the biofilm that had developed in each well was washed twice with 200 μL of distilled water and then dried for 45 min. One hundred μL of 0.4 % crystal violet were added to each well for 30–45 min. After this procedure, the wells were washed four times with distilled water and immediately discolored with 200 μL of 95 % ethanol. After 45 min, 100 μL of discolored solution was transferred to a well of a new plate and the crystal violet measured at 570 nm in a microplate reader (Tecan). The amount of biofilm formed was measured comparing the absorbance values of the compound-treated wells versus untreated control wells. Biofilm formation bioassays were performed in triplicate in at least three individual experiments for each concentration.

### Effect of diselenides on biofilm dispersion

Biofilms were grown on the inside surface of a 96-well microtiter plate. Biofilms grown, as described above, were then treated with three different concentrations of diselenides as dispersion inducer or just the diluent, at the same concentrations used to dilute diselenides as a control, and incubated at 37 °C for a further 24 h. The positive control for Gram-positive and Gram-negative bacteria was gentamicin, and fluconazole for *C. albicans*.

Afterward, the biofilm mass was quantified by crystal violet assay. Biofilm dispersal bioassays were performed in triplicate in at least three individual experiments for each concentration.

### Antioxidant activity

The effect of antioxidant compounds on DPPH radical has been detected by spectrophotometer analysis. The reduction of the radical by hydrogen atom transfer from a hydrogen donor (antioxidant) with the formation of the hydrazine DPPH-H causes a change in the color of the solution from violet to pale yellow [23, 24]. The percentage of DPPH radical scavenging ratio of each diselenides was assayed by di(phenyl)-(2,4,6-trinitrophenyl) iminoazanium (DPPH) assay as previously described [25]. DPPH is a stable free-radical molecule at room temperature. In the presence of antioxidant molecules, which can donate hydrogen, DPPH is reduced giving a variation of colour evaluable by spectrophotometry. The reaction mixture consisted of a 100 μl of sample and 100 μl of DPPH radical solution in ethanol (50 mg/L). The change in colour (from deep violet to light yellow) of DPPH was determined at 517 nm after 30 min of reaction using a microplate reader (Tecan). The mixture of ethanol and sample was used as blank. The control solution was prepared by mixing ethanol and DPPH radical solution. Ascorbic acid was used as a positive control. The percentage of DPPH radical scavenging ratio [26] was calculated according the following formula:

$$ \%\ \mathrm{DPPH}\ \mathrm{radical}\ \mathrm{scavenging}\ \mathrm{ratio} = \left[1\hbox{-} \left(\mathrm{Abs}\ \mathrm{sample}\hbox{-} \mathrm{Abs}\ \mathrm{blank}\right)/\mathrm{Abs}\ \mathrm{control}\right] \times 100. $$

### Cell viability assay

Cytotoxicity was tested by the determination of the cell ATP level by ViaLight® Plus Kit (Lonza). This method is based upon the bioluminescent measurement of ATP that is present in all metabolically active cells. The bioluminescent method utilizes an enzyme, luciferase, which catalyzes the formation of light from ATP and luciferin. The emitted light intensity is linearly related to the ATP concentration and is measured using a luminometer. All diselenides were tested on a human cervix adenocarcinoma epithelial HeLa cell line (HeLa), human dermis fibroblast (HuDe) and human skin keratinocytes (NCTC2544) cells, which were grown in RPMI 1640 supplemented with 10 % heat-inactivated foetal calf serum, 10,000 units penicillin and 10 μg streptomycin/ml overnight to confluence. Monolayer cells were treated for 24 h at 37 °C with scalar concentrations of tested compounds (0, 0.22, 0.45, 0.9, 1.8, 3.9, 7.8, 15.6, 31.25, 62.5, 125, 250 mg/L). After incubation, the plates were left at room temperature to cool for 10 min and then the Cell Lysis Reagent was added to each well to extract ATP form the cells. Next, after 10 min, the AMR Plus (ATP Monitoring Reagent Plus) was added and after 2 more minutes the luminescence was read using a microplate luminometer (TECAN). Results are expressed as CC_50_. The 50 % cytotoxic concentration (CC_50_) was defined as the concentration required to reduce the live cell number by 50 %, compared to the untreated controls.

### Statistical analysis

All experiments were performed in triplicate in at least three different experiments. Data were expressed as mean ± SD. Differences between diselenide-treated biofilm and untreated biofilm were compared using the Student’s t-test (two-tailed). *P-values of < 0.05 were considered significant.

## Results

### Antimicrobial activity of diselenides

The screening of diselenides as antimicrobial drugs versus different microorganisms is shown in Table 2. The results indicated that MIC of compounds 9 and 10 showed a moderate antimicrobial activity against *S. epidermidis*, *S. pyogenes* and *C. albicans*; whereas compounds 10_d_ and 10_e_ demonstrated considerable antibacterial activity versus *S. epidermidis* and *S. pyogenes.*

**Table 2:** Minimal Inhibitory Concentration (MIC) of diselenides against different microorganisms

MIC (mg/L)	9	10	11	10c	10d	10e	Positive control^a^
*Staphylococcus aureus*	>250	125	250	>250	>250	31.25	7.8
*Staphylococcus epidermidis*	15.62	3.9	>250	>250	7.8	7.8	0.18
*Streptococcus pyogenes*	15.62	7.8	31.25	>250	15.62	31.25	2.19
*Streptococcus pneumoniae*	>250	125	>250	250	>250	>250	0.45
*Pseudomonas aeruginosa*	>250	>250	>250	>250	250	>250	1.5
*Candida albicans*	31.25	62.5	125	>250	>250	>250	0.25

MIC was evaluated by standardized CLSI methods

^a^The positive control for Gram-positive and Gram-negative bacteria was gentamicin and Fluconazole for *Candida albicans*

**Fig. 1:**
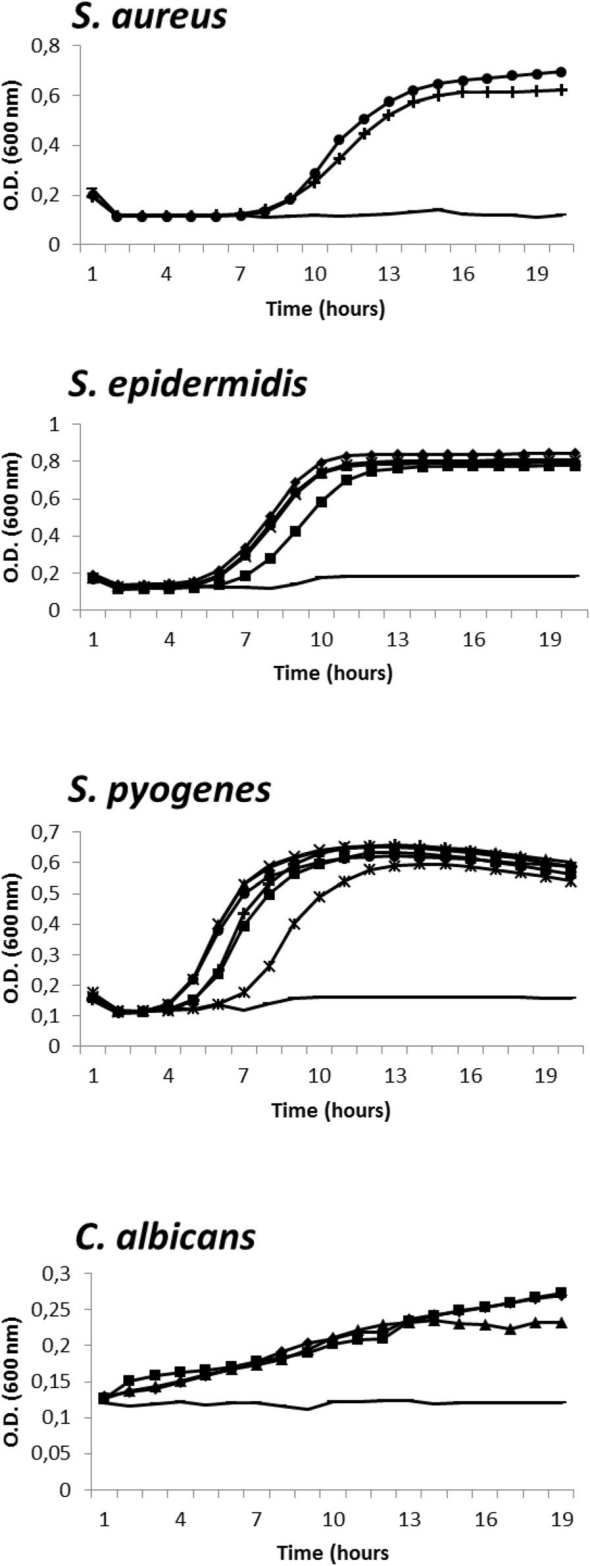
Growth curve of *S. epidermidis, S. pyogenes, P. aeruginosa* and *C. albicans* in the presence of diselenides. Concentrations of diselenides 0.2xMIC or 0.5xMIC were tested for bacteria or fungus respectively. The positive control for Gram-positive and Gram-negative bacteria was gentamicin, and fluconazole for *Candida albicans* (untreated microorganisms: filled circles, 9: filled squares, 10: filled triangles, 11:*, 10_c_: filled rhombuses, 10_d_: X, 10_e_: +, positive control -). Data are expressed as a mean of six replicates from two independent experiments

The kinetics of microbial growth were investigated to identify the right concentration of compounds to use in the antibiofilm test to keep out direct antimicrobial properties. The inhibitory effects of diselenides on the growth of *S. epidermidis, S. pyogenes, P. aeruginosa* and *C. albicans* are reported in Fig. 1. At the concentration of 2xMIC and 1xMIC, all the compounds inhibited the growth of all microorganisms (data non shown), while at 0.2xMIC for bacteria and 0.5xMIC for *C. albicans* the growth curves observed were not significantly different to those obtained for untreated bacterial cultures, suggesting that diselenides did not affect microbial division at the concentrations tested.

### Antibiofilm activity

**Fig. 2:**
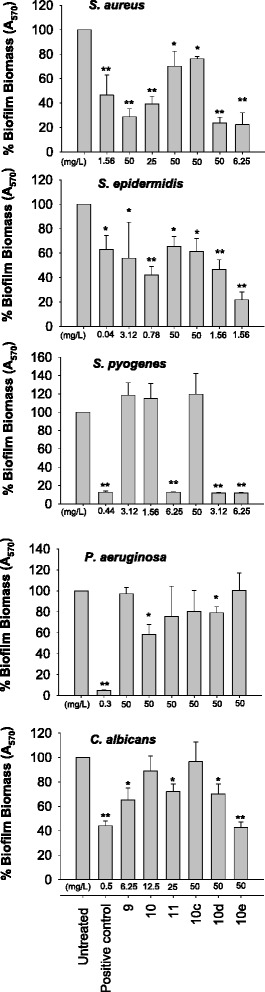
The effect of diselenides on biofilm formation. *S. aureus, S. epidermidis, S. pyogenes, P. aeruginosa,* and *Candida albicans* were inoculated into a 96-well plate containing diselenides and incubated for 24 h. The concentration of the different diselenides used for each microorganism is indicated under the X-axis of the corresponding histogram. Biofilm biomass was quantified by crystal violet assay (absorbance 570 nm). Data represent the mean ± SD of 2 or more independent experiments performed in triplicate. *P < 0.05, **P < 0.01 (treated microorganisms versus untreated cells)

To analyze in depth the antimicrobial properties of diselenides, we examined the ability of *S. aureus*, *S. epidermidis*, *S. pyogenes*, *P. aeruginosa* and *C. albicans* to form biofilm in the absence or presence of organoselenium compounds. Biofilm formation was measured by determining the mass of biofilm using crystal violet staining. Biofilms were grown in static conditions in the presence of diselenides at concentrations lower than MIC. In particular, we used a concentration of 0.2 × MIC for the bacterial strains tested and 0.5 × MIC for *C. albicans*, which resulted ineffective on microbial growth (Fig. 1). For diselenides with a MIC ≥ 250 mg/L, we used the concentration of 50 mg/L. All diselenides showed a good antibiofilm activity against the Gram-positive bacteria *S. aureus* and *S. epidermidis*, while diselenides 11, 10_d_ and 10_e_ were effective against *S. pyogenes*. Diselenides resulted less active against *P. aeruginosa*, in fact only compound 10 and 10d reduced anti-biofilm activity by 20 % and 40 % respectively. Finally, diselenides 9, 11, 10_d_ and 10_e_ showed anti-biofilm activity against the yeast *C. albicans* (Fig. 2).

**Fig. 3:**
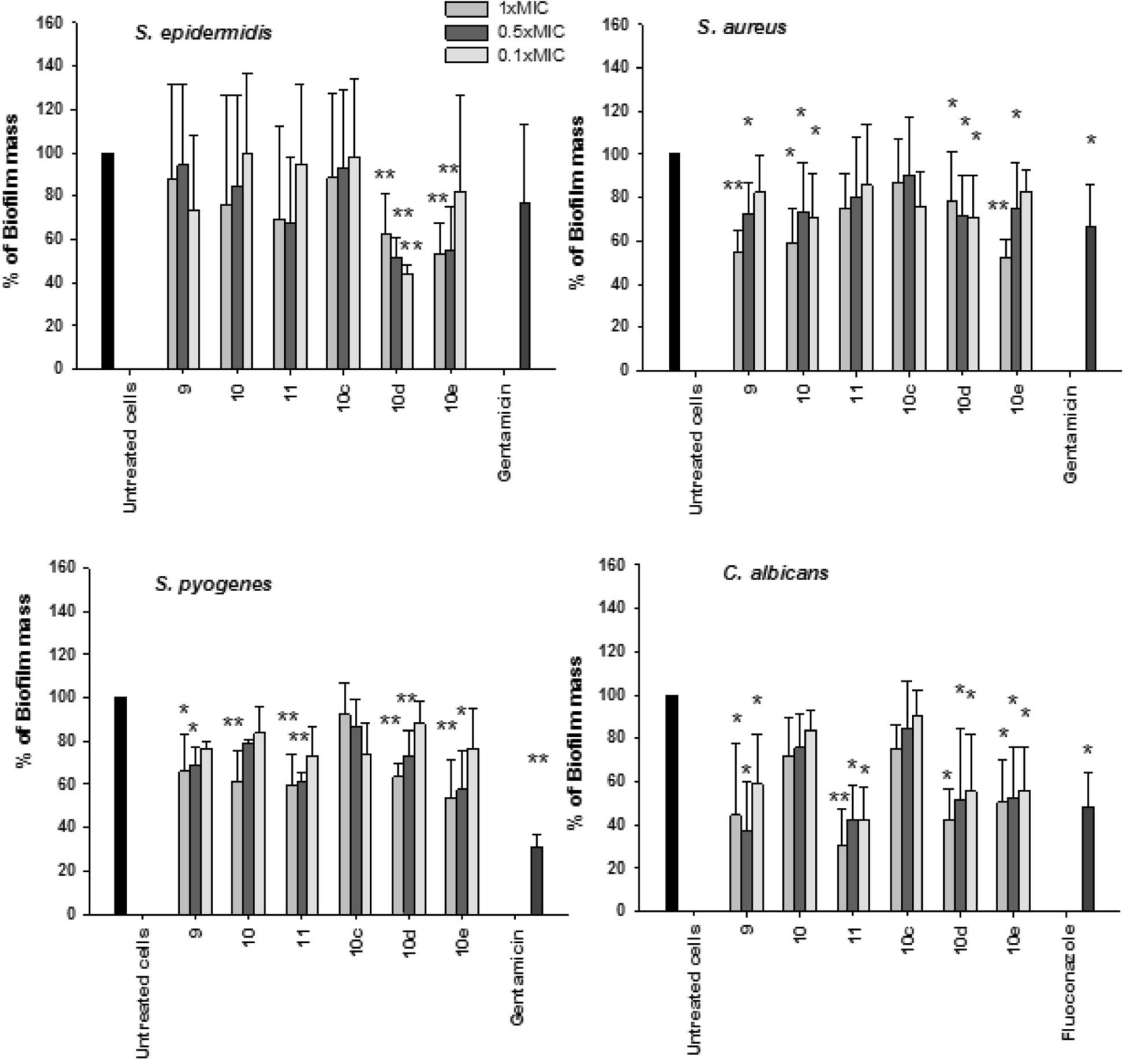
The effect of diselenides on biofilm dispersion. *S. aureus, S. epidermidis, S. pyogenes, P. aeruginosa,* and *C. albicans* were inoculated into a 96-well plate and incubated for 24 h. After incubation three different concentrations of diselenides (1 x MIC; 0.5 × MIC; 0.1 × MIC) were added to preformed biofilm. The plates were then incubated for 24 h. Biofilm biomass was quantified by crystal violet assay (absorbance 570 nm). Data represent the mean ± SD of 2 or more independent experiments performed in triplicate. *P < 0.05, **P < 0.01 (treated microorganisms versus untreated cells)

In parallel experiments, the ability of diselenides to disperse preformed biofilm was assayed against Gram-positive bacteria and the yeast *C. albicans*. Different concentrations of diselenides corresponding to the 1 x MIC, 0.5 x MIC and 0.1 × MIC were added on preformed biofilm and their effect on dispersal was determined after 24 h of incubation. For diselenides with a MIC ≥ 250 mg/L we tested the concentrations 250, 125 and 25 mg/L. The results in Fig. 3, showed that diselenides 10_d_ and 10_e_ were able to reduce the biofilm mass of *S. epidermidis,* compound 10_d_ was able to reduce biofilm at all concentrations tested; of note is that this compound was able to reduce the biofilm mass at one tenth of the MIC confirming that the antibiofilm effect is not due to a direct killing effect; instead compound 10_e_ was active in the amount of 1xMIC and one half of MIC. Biofilm compounds 10 and 10_d_ showed inhibitory activity against *S. aureus* at all doses tested, while diselenides 9 and 10_e_ were active up to 0.5 MIC. Diselenides 9, 10, 11 10_d_ and 10_e_ were able to decrease the preformed biofilm *S. pyogenes* at concentrations of 1 and 0.5 × MIC. Finally, *C. albicans* preformed biofilm was dispersed by compounds 9, 11, 10_d_ and 10_e_ at all concentrations tested (Fig. 3). Up to this point, compound 10_d_ was the most active in this series of diselenides.

### Antioxidant activity

Non-healing wounds in humans have shown high oxidative and nitrosative stress [27–29]. Exacerbation of oxidative stress and biofilm-forming bacteria are critical for the initiation of chronicity [27]. In the last few decades, the biological antioxidant property of new synthetic organic selenium compounds has been reported [30–35]. The antioxidant mechanism of action of organoselenium compounds depends on the cellular environment. These agents do not change the redox balance themselves, but their activities depend on the cellular redox state in which they are placed. Mounting evidence suggests that regular uptake of antioxidants is required to scavenge ROS (Radical Oxygen Species) and RNS (Radical Nitrogen Species) [36, 37].

**Fig. 4:**
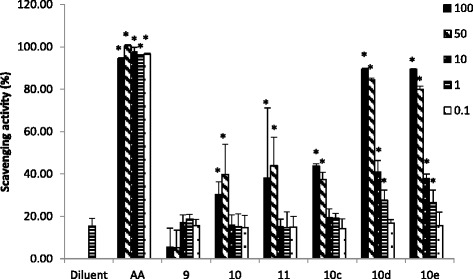
Antioxidant activity of diselenides. Antioxidant activity was determined by DPPH reduction; diselenide activity was determined and compared to the standard ascorbic acid (AA). Results are expressed as DPPH scavenging activity. Data represent the mean ± SD of 2 or more independent experiments performed in triplicate. Differences between antioxidant activity of diselenides and diluent were compared using the Student’s t-test (two-tailed). *P < 0.05, (diselenides effect versus diluent effect)

In order to develop strategies to reduce redox stress and inhibit biofilm formation to restore wound tissue, we tested the antioxidant activity of diselenides at three different concentrations (100-50-10-1-0.1 mg/L) by DPPH assay. As negative control, the diluent used in preparing stock solution of diselenides was used; methanol was added to the sample and its antioxidant activity was determined. The results shown in Fig. 4 prove that compounds 9, 10, 11 and 10_c_ had no antioxidant activity at concentrations of 0.1, 1 and 10 mg/L and a weak activity at 50 and 100 mg/L. Furthermore, diselenides 10_d_ and 10_e_ exhibited a moderate dose scavenging activity at 10 mg/L and a very good antioxidant property at 50 and 100 mg/L as compared to that observed with the positive control ascorbic acid.

### Biocompatibility of Diselenides

For tissue engineering materials or drug carrier application, diselenides must be non-toxic and biocompatible. HeLa human epithelial cells, HuDe human dermal fibroblast cells and NCTC2544 human keratinocyte cells were exposed to scalar concentrations of diselenides before ATP level measurement. The CC_50_ obtained for all diselenides tested is reported in Table 3. Diselenides exhibited different cytotoxicity toward different cell lines. Compounds 11, 10_d_ and 10_e_ were highly toxic against HeLa cells, while diselenide 10_c_ showed mild toxicity and compounds 9 and 10 had low toxicity. Human fibroblast HuDe showed a slight reduction in vitality in the presence of compounds 10, 10_d_ and 10_e_, while diselenides 9, 10_c_ were mildly toxic and compound 11 showed a strong toxicity. Finally, for human keratinocytes NCTC2544, the CC_50_ of compounds 10_c_ and 10_e_ was ≥ 250 mg/L; diselenides 10, and 10_d_ showed moderate toxicity while compounds 9 and 11 were toxic.

**Table 3:** Cytotoxicity of diselenides against Hela, HDF and NCTC2544 cell lines

CC_50_ (mg/L)	9	10	11	10_c_	10_d_	10_e_
*Hela*	123.69	113.69	10.92	32.16	19.25	9.71
*HDF*	55.02	135.86	8.36	35.29	112.93	>250
*NCTC2544*	14.62	44.61	18.45	250.00	23.84	>250

Cytotoxicity was tested by the determination of the cell ATP level by a bioluminescent method after 24 h of incubation. CC_50_ is the concentration required to reduce the live cell number by 50 % compared to the untreated controls. The CC_50_ values are reported as the means from two independent experiments performed in duplicate

## Discussion

Given the role that pathogenic biofilms play in impairing the healing of chronic wounds, preventing biofilm formation is fundamental for faster and more effective treatment. When the biofilm is well established, microorganisms inside the matrix will exhibit resistance to killing by the host immune system and antimicrobials. In the past two decades a variety of organoselenium compounds have been tested against bacteria, fungi, algae and viruses. Most of them have shown a good activity with respect to the antimicrobial drugs in current use [14]. In particular, organoselenium coating on cellulose was able to inhibit *P. aeruginosa* and *S. aureus* biofilm formation [38, 39]. The antifungal property of biphelyl diselenide (PhSe)_2_ against different species of *Candida* has been reported by Loreto ES et al. [16]. The values for *C. albicans* reported in their study are similar to those obtained in our experimental conditions.

The antibiofilm activity of other organoselenium compounds against *P. aeruginosa* [36] and *S. aureus* has been tested in in vivo and in vitro studies [36, 37]. We tested six diselenides for their activity against preformed biofilms as well during biofilm formation. All compounds were able to reduce the biofilm formation of Gram-positive Staphylococci. Diselenides 11, 10_d_ and 10_e_ inhibited the formation of *S. pyogenes* biofilm; while, only compounds 10 and 10_d_ were active against the Gram-negative *P. aeruginosa*. This effect is probably due to the different structures of the microbial cell walls. Dispersal of the biofilm test showed that *S. epidermidis* biofilm is dispersed by diselenides 10_c_ and 10_d_, while *S. aureus* biofilm is partially reduced by compounds 9, 10 10_d_ and 10_e_; dispersal biofilm of *S. pyogenes* has been observed in the presence of all compounds except 10_c_. Compounds 9, 10, 10_d_ and 10_e_ were able to inhibit and to disperse the biofilm of *C. albicans.* The overall results evidenced that diselenides 10_d_ and 10_e_ showed the best antibiofilm activity both in biofilm formation and dispersion. The antimicrobial activity of 2,2’-dithienyl diselenide against bacteria and *C. albicans* has been recently reported; the mechanism of action suggested was the pro-oxidant activity [15]. However, the anti-oxidant activity of diselenides has been demonstrated [23]. This apparent incongruity is consistent with the structures of diselenides 10_d_ and 10_e_; in fact the amide group, by a nonbonding interaction, enhances the electrophilicity of the selenium atom, by activating the oxidation of the dichalcogenide bond. This confers radical scavenger properties to the structure but, at the same time, the oxidative cleavage of the Se-Se bond leads to the formation of strongly oxidant intermediates (e.g. selenenic and seleninic species). If not readily reduced by a glutathione mediated reaction (like in the GPx catalytic cycle), such intermediates can be responsible for the pro-oxidant activity.

The antioxidant activity of new selenide compounds has been reported; monoselenides showed a weaker effect compared to diselenides [39–41]. Novel nitrogen-containing diselenides can act against oxidative stress through a glutathione peroxidase-like activity [19]. As *S. aureus*, *S. epidermidis* and *S. pyogenes* are the most common isolates among wound infections [8], we can assume that diselenides have a potential for development as therapeutic antimicrobials for wound infections. Moreover, diselenides affected cell vitality according to the cell line used; fibroblast HuDe cells resulted more resistant than human keratinocytes to compounds 9, 10, 10_d_ and 10_e_, while NCTC2544 cells are less sensitive to diselenide 10_c_. The different cytotoxic activity is likely due to the intrinsic differences among cell lines and multiple factors such as cell permeability and macromolecular target binding.

## Conclusions

In conclusions, considering that compound 10_e_ was the most biocompatible against fibroblast and keratinocytes, the antioxidant activity of compounds 10_d_ and 10_e_ and the antibiofilm properties of compounds 10_d_ and 10_e_ against *S. aureus*, *S. epidermidis*, *S. pyogenes* and *C. albicans,* diselenides 10_d_ and 10_e_ could be very good candidates for the development of new therapeutic applications for acute wound infections as well as chronic skin diseases such as diabetic foot ulcers and venous stasis ulcers may be possible.

## Abbreviations

AA: Ascorbic acid
AMR: ATP Monitoring Reagent
ATP: Adenosine triphosphate
CC_50_: 50 % cytotoxic concentration
CLSI: Clinical and Laboratory Standards Institute
DPPH: di(phenyl)-(2,4,6-trinitrophenyl) iminoazanium
GPx: Glutathione peroxidase
HeLa: HeLa cell line
HuDe: human dermis fibroblast
MHB: Muller Hinton Broth
MIC: Minimum inhibitory concentration
MOPS: 3-(N-morpholino)propanesulfonic acid
NCTC2544: Human skin keratinocytes
RNS: Radical nitrogen species
ROS: Radical oxygen species
RPMI: Roswell Park Memorial Institute
SD: Standard deviation
TSA: Tryptic soy agar
TSB: Tryptic soy broth
